# Effects of the combination of camptothecin and doxorubicin or etoposide on rat glioma cells and camptothecin-resistant variants

**DOI:** 10.1054/bjoc.2001.2027

**Published:** 2001-09

**Authors:** V Pavillard, D Kherfellah, S Richard, J Robert, D Montaudon

**Affiliations:** Laboratoire de Pharmacologie des Médicaments Anticancéreux, Institut Bergonié et Université Victor Segalen Bordeaux 2, 180 rue de Saint-Genès, Bordeaux-cedex, 33076, France

**Keywords:** topoisomerase inhibitors, drug combinations, camptothecin resistance

## Abstract

From the rat C6 glioma cell line in culture, we selected camptothecin-resistant variants by growth in the presence of increasing amounts of this drug (C6_CPT10_, C6_CPT50_ and C6_CPT100_, growing respectively with 10, 50 and 100 ng ml^–1^camptothecin). The degree of resistance to camptothecin ranged between 15-fold (C6_CPT10_) and 30-fold (C6_CPT50_and C6_CPT100_). The C6_CPT10_cell line presented a collateral sensitivity to etoposide (3.6-fold), while the C6_CPT50_ and C6_CPT100_ cell lines were cross-resistant to etoposide (1.8-fold) The resistant lines were characterised by a two-fold reduced content and catalytic activity of topoisomerase I, and C6_CPT50_ and C6_CPT100_ presented a significant increase in topoisomerase IIα content and catalytic activity and a marked overexpression of P-glycoprotein. We explored the cytotoxicity of combinations of a topoisomerase I inhibitor (camptothecin) and a topoisomerase II inhibitor (doxorubicin or etoposide) at several molar ratios, allowing the evaluation of their synergistic or antagonistic effects on cell survival using the median effect principle. The simultaneous combination of camptothecin and doxorubicin or etoposide was additive or antagonistic in C6 cells, slightly synergistic in the C6_CPT10_ line and never more than additive in the C6_CPT50_ and C6_CPT100_ cell lines. The sequential combination of doxorubicin and camptothecin gave additivity in the order camptothecin → doxorubicin and antagonism in the order doxorubicin → camptothecin. Clinical protocols combining a topoisomerase I and a topoisomerase II inhibitor should be considered with caution because antagonistic effects have been observed with combinations of camptothecin and doxorubicin.© 2001 Cancer Research Campaign http://www.bjcancer.com


*British Journal of Cancer* (2001) **85**(7), 1077–1083
© 2001 Cancer Research Campaign
doi: 10.1054/ bjoc.2001.2027, available online at http://www.idealibrary.com on IDEAL

http://www.bjcancer.com

### Effects of the combination of camptothecin and doxorubicin or etoposide on rat glioma cells and camptothecin-resistant variants

**V Pavillard, D Kherfellah, S Richard, J Robert and D Montaudon**

Laboratoire de Pharmacologie des Médicaments Anticancéreux, Institut Bergonié et Université Victor Segalen Bordeaux 2, 180 rue de Saint-Genès 33076 Bordeaux-cedex, France

**Summary** From the rat C6 glioma cell line in culture, we selected camptothecin-resistant variants by growth in the presence of increasing amounts of this drug ($C6_{CPT10}$, $C6_{CPT50}$ and $C6_{CPT100}$, growing respectively with 10, 50 and 100 ng ml$^{-1}$ camptothecin). The degree of resistance to camptothecin ranged between 15-fold ($C6_{CPT10}$) and 30-fold ($C6_{CPT50}$ and $C6_{CPT100}$). The $C6_{CPT10}$ cell line presented a collateral sensitivity to etoposide (3.6-fold), while the $C6_{CPT50}$ and $C6_{CPT100}$ cell lines were cross-resistant to etoposide (1.8-fold) The resistant lines were characterised by a two-fold reduced content and catalytic activity of topoisomerase I, and $C6_{CPT50}$ and $C6_{CPT100}$ presented a significant increase in topoisomerase IIα content and catalytic activity and a marked overexpression of P-glycoprotein. We explored the cytotoxicity of combinations of a topoisomerase I inhibitor (camptothecin) and a topoisomerase II inhibitor (doxorubicin or etoposide) at several molar ratios, allowing the evaluation of their synergistic or antagonistic effects on cell survival using the median effect principle. The simultaneous combination of camptothecin and doxorubicin or etoposide was additive or antagonistic in C6 cells, slightly synergistic in the $C6_{CPT10}$ line and never more than additive in the $C6_{CPT50}$ and $C6_{CPT100}$ cell lines. The sequential combination of doxorubicin and camptothecin gave additivity in the order camptothecin → doxorubicin and antagonism in the order doxorubicin → camptothecin. Clinical protocols combining a topoisomerase I and a topoisomerase II inhibitor should be considered with caution because antagonistic effects have been observed with combinations of camptothecin and doxorubicin. © 2001 Cancer Research Campaign http://www.bjcancer.com

**Keywords**: topoisomerase inhibitors; drug combinations; camptothecin resistance

The nuclear enzymes DNA topoisomerase I (top 1) and DNA topoisomerase II (top 2) represent important primary targets for the action of anticancer drugs (Liu, 1989). These enzymes are able to induce and religate DNA strand breaks in order to allow multiple topological modifications of DNA such as relaxation of the supercoiled molecules and decatenation and unknotting of intermingled fragments (Andersen et al, 1994). Relaxation is required when the RNA and DNA polymerases operate, in order to relieve the torsional constraints of intertwined DNA strands during the operations of transcription and replication. Decatenation is required during mitosis, for the segregation of sister chromatides in the daughter cells. Top 1 induces DNA single-strand breaks and is mainly involved in DNA relaxation, whereas top 2 induces DNA double-strand breaks, creating a gap through which another DNA double strand can cross the first one ('strand passing'): therefore, the topological activities of top 2 cannot be ensured by top 1, whereas top 2 is able to provide DNA relaxation (Nitiss, 1994).

Many drugs classes have been shown to interfere with DNA topoisomerases: aminoacridines (amsacrine), epipodophyllotoxins (etoposide) and anthracyclines (doxorubicin) interfere with the religation reaction of top 2, whereas camptothecin and its water-soluble analogues (irinotecan, topotecan) interfere with the same step of top 1 action. In both cases, the drugs stabilise the topoisomerase–DNA complex, which is often called the 'cleavable complex', transforming the transient breaks into permanent breaks, which is considered by the cell as a lethal lesion leading to cell death (Osheroff et al, 1994; Pommier et al, 1994). Other drugs have been shown to interfere with other catalytic steps of the DNA-topoisomerase action and present some antiproliferative properties, but none of them has yet been approved as an anticancer drug.

One of the mechanisms of resistance to topoisomerase-interfering drugs consists in the alteration of the target itself (Andoh and Okada, 1994; Robert and Larsen, 1998). A frequent feature characterising cell lines which have been rendered resistant to such drugs is the strong decrease in drug-induced cleavable complex formation, as compared to the corresponding sensitive cell lines. This may be due either to a quantitative defect in topoisomerase expression, which has been shown to occur at the mRNA and at the protein levels, or to a qualitative alteration of the topoisomerase amino acid sequence leading to reduced cleavable complex formation in the presence of drugs.

In both cases, it has been postulated that the reduced catalytic activity following the quantitative or qualitative enzyme alteration could be compensated for by the topoisomerase of the other class, since the actions of both enzymes are partially overlapping (Tan et al, 1989). Indeed, an increase in top 2 activity has been shown to occur in cell lines resistant to camptothecin, a top 1 inhibitor (Sugimoto et al, 1990), and a collateral sensitivity of camptothecin-resistant cells to doxorubicin has been observed (Oguro et al,

*Received 11 January 2001*
*Revised 18 June 2001*
*Accepted 20 June 2001*

*Correspondence to:* J Robert

1990). However, increased top 1 activity has not yet been noticed in cell lines resistant to etoposide or doxorubicin. One can hypothesise, therefore, that the concomitant use of top 1 and top 2 inhibitors may have synergistic effects in cell lines resistant to top 1 inhibitors. We wanted to explore this possibility in a cellular model developed and studied in our laboratory and its variants resistant to camptothecin, in order to determine whether clinical applications could be implied from these in vitro studies.

### MATERIALS AND METHODS

### Cell culture

The cell lines used in this study originate from a chemically induced rat glioblastoma cell line, C6 (Benda et al, 1968), selected because of its properties to generate orthotopic and heterotopic tumours in Wistar rats. We have selected two variants of this line by continuous in vitro growth in the presence of stepwise increasing concentrations of camptothecin: these variants grow in the continuous presence of 10 ($C6_{CPT10}$), 50 ($C6_{CPT50}$) and 100 ($C6_{CPT100}$) ng of camptothecin per ml of medium, respectively. Cells were routinely grown as monolayers in Petri dishes with Dulbecco's modified Eagle's medium (DMEM) supplemented with 10% fetal calf serum and antibiotic mixture, in an humidified atmosphere in a $CO_2$ incubator at 37˚C. The doubling times of the cell populations were as follows: C6: 18 h; $C6_{CPT10}$: 20 h; $C6_{CPT50}$: 30 h; $C6_{CPT100}$: 28 h.

### Drugs and chemicals

Doxorubicin and etoposide were obtained as chemical formulations from Pharmacia & Upjohn and Novartis, respectively. Camptothecin was purchased from Sigma Chimie (Saint-Quentin-Fallavier, France). Stock solutions were prepared periodically in distilled water or dimethylsulfoxide (for camptothecin) and stored at –20˚C. Chemicals were of the highest commercially available quality. Top 1 and top 2α standards, *Trypanosoma* kinetoplast DNA, and the rabbit antibodies against the human enzymes top 1 and top 2α were obtained from Topogen (Columbus, OH, USA). The C219 antibody against P-glycoprotein was obtained from Centocor (Leiden, The Netherlands). Phagemid pBluescript II KS was purchased from Promega (Charbonnières-les-Bains, France).

### Cytotoxicity

Adequate numbers of cells were seeded in 10 $cm^2$ Petri dishes and grown for at least one doubling time. Drugs were then added to the culture medium and left for the duration of one doubling time in contact with the cell monolayer. After removal of the drug-containing medium, the cell monolayers were rinsed twice with saline solution and cells were allowed to further grow with fresh medium for the duration of two more doubling times, such that the cells remained in exponential phase of growth during the entire process. Cells were then recovered after trypsinisation and counted in a Coulter Counter ZM (Coultronics, Margency, France). Each cell line was characterised by its $IC_{50}$ value to a given drug, i.e. the drug concentration reducing cell number by 50% as compared to drug-free controls. $IC_{50}$s were calculated after at least 3 independent experiments performed in triplicate.

In order to evaluate possible synergistic or antagonistic effect of top 1 or top 2 inhibitors, we used mixtures of camptothecin and either doxorubicin or etoposide over a wide range of concentrations, at 3 different dose ratios for simultaneous combinations, and at one dose ratio for sequential combinations. The dose ratios selected corresponded to the ratios of the $IC_{50}$ of individual drugs. The data were analysed according to the median effect principle, as described by Chou and Talalay (1984). Combination indices were calculated on a microcomputer using the software of Chou and Chou (1987). Combination index values either lower or higher than 1 indicate synergistic or antagonist effect, respectively.

### Immunoblot analysis of topoisomerase II

Nuclear extracts were prepared from about $1 \times 10^8$ exponentially growing cells as already described (Montaudon et al, 1997). Briefly, nuclei were prepared after lysis by Dounce homogenisation and centrifugation at 1500 ***g*** for 5 min at 4˚C. Nuclear proteins were extracted for 60 min at 4˚C. DNA and nuclear fragments were precipitated by centrifugation at 19 000 ***g*** for 30 min at 4˚C. Supernatant protein concentration was determined with the Bradford assay using bovine serum albumin as a standard. We used 1 M NaCl extracts for immunoblotting and 0.35 M NaCl extracts for decatenation or relaxation assays.

Proteins were subjected to electrophoresis according to Laemmli (1970) at 80 V overnight. Positive controls of human top 1 or top 2α (Topogen) were run simultaneously to locate the position of the proteins and the reactivity of the antibodies. Proteins were transferred to Immobilon-P membranes (Millipore) using an electroblotting device (Milliblot, Millipore, Saint-Quentin-en-Yvelines, France) at 2.5 $mA/cm^2$ for 1 h. Membranes were then incubated for 2 h at room temperature either with a rabbit polyclonal antibody against top 2α (Topogen) at a 1:750 dilution, or with a rabbit polyclonal antibody against top 1 (Topogen), at a 1:2000 dilution. Blots were then treated either with an alkaline phosphatase-labelled goat antibody against rabbit immunoglobulins (Dako, Trappes, France), at a 1:300 dilution, or with a horseradish peroxidase-labelled monkey antibody against rabbit immunoglobulins (Amersham Pharmacia Biotech, Orsay, France) at a 1:4000 dilution. Immunoreactive bands were visualised either by incubation for 5–10 min in 5-bromo-4-chlore-indolyl phosphate and nitroblue tetrazolium (BCIP tablets from Sigma Chimie), or with the peroxidase substrate (Lumigen PS-3 acridan, ECL Plus, Amersham Pharmacia). Spots were quantified by densitometric scanning with the Densylab software (Microvision Instruments).

### Evaluation of the catalytic activities of topoisomerase II and topoisomerase I

Top 2 activity was evaluated by decatenation of a catenated DNA substrate originating from *Trypanosoma* kinetoplasts (kDNA, Topogen) into various relaxed DNA forms as already described (Montaudon et al, 1997). The reaction was initiated by the addition of 0.35 M NaCl nuclear extracts and stopped after a 20-min incubation at 37˚C by adding a denaturing solution. The samples were then electrophoresed on a 1% agarose gel for 45 min at 80 V. A positive control for the reaction was a decatenated kDNA marker (Topogen). DNA was visualised under ultraviolet light and the various DNA forms were quantified by densitometric scanning. The catalytic activity of topoisomerase II was evaluated as the amount of ng kDNA decatenated per μg protein in the nuclear extract.

 © 2001 Cancer Research Campaign

Top 1 activity was determined using the DNA relaxation assay previously described by Liu and Miller (1981) with some modifications. Different concentrations of 0.35 M NaCl nuclear extracts and 1 µg of pBKS IIKS supercoiled DNA were mixed on ice in a final volume of 20 µl. The reaction mixtures were incubated at 37˚C for 30 min and the reaction stopped on ice. The samples were then submitted to electrophoresis in 1% agarose gel at 80 V for 1 hour. DNA was visualised under UV light and quantified by densitometric scanning. The catalytic activity of topoisomerase I was evaluated as the amount of ng DNA relaxed per ng protein in the nuclear extract.

### Immunoblot blot analysis of P-glycoprotein

Electrophoresis of whole cell lysates (400 µg proteins per lane) and protein transfer on Immobilon-P membranes were carried out as already described (Huet et al, 1992). The blots were incubated with C219 monoclonal antibody at 4˚C overnight. The membranes were then incubated with alkaline phosphatase-conjugated rabbit anti-mouse immunoglobulins and developed using BCIP substrate. Spots were quantified as already mentioned.

### Evaluation of DNA damage in C6 cells

Analysis of drug-induced DNA strand breaks was performed using the filter alkaline elution technique (Kohn, 1991) as already described (Montaudon et al, 1997). The cells were labelled with [methyl-$^3$H]thymidine (0.1 mCi ml$^{-1}$ medium, Amersham Pharmacia Biotech), then treated with different drug concentrations for 2 h at 37˚C. Cell pellets were resuspended and layered onto polycarbonate membranes of 25 mm diameter and 2 mm pore size (Nucleopore, purchased from Schumacher-DMF, Gonesse, France). The cells were lysed in situ for 1 h in the presence or absence of 0.5 mg ml$^{-1}$ proteinase K (Roche, Meylan, France), and the DNA on the filter was eluted with tetrapropylammonium hydroxide (Kodak, purchased from Touzart et Matignon, Courtabœuf, France), pH 12.5. Elution was carried out at 0.044 ml min$^{-1}$ for a total of 15 h. The radioactivity on the filters and collected fractions was determined in a liquid scintillation spectrometer. It was then possible to calculate the fraction of DNA retained on the filter versus the elution time. The slopes from each elution profile were used to determine the elution rate constant for a given drug concentration. The radiation-induced elution rate constants had previously generated standard curves (Montaudon et al, 1997) allowing the determination of the radiation equivalence of each drug concentration. Results of drug-induced DNA damage were expressed as equivalent radiation dose in Gray (Gy).

### HPLC evaluation of camptothecin cellular accumulation

Evaluation of camptothecin concentration in cells was performed by HPLC as already described (Rivory and Robert, 1994). Cell extracts were obtained in methanol/acetonitrile (50/50, v/v) containing 1% HCl. Separation was carried out on a C-18 reversed-phase column (Nova-Pak, Radial Pak, Waters, Saint-Quentin-en-Yvelines, France) with a mobile phase consisting of a mixture of acetonitrile and 0.075 M ammonium acetate buffer, pH 6.0, containing 5 mM tetrabutylammonium phosphate (Pic-A, Waters). This mobile phase was delivered isocratically at a flow rate of 1.5 ml min$^{-1}$ with a Spectra Systems P4000 XR pump (Thermo Quest, Les Ulis France). Fluorometric detection was carried out with excitation and emission wavelengths set at 355 and 515 nm respectively, using the Spectra Systems FL 3000 (Thermo Quest) detector. Peaks were quantified by reference to a standard calibration curve obtained by spiking known amounts of drugs in untreated cell extracts, thanks to the PC1000 software (Thermo Quest).

### RESULTS

### Characterisation of camptothecin-resistant cell lines

We selected the three C6 sublines, $C6_{CPT50}$ and $C6_{CPT100}$, by continuous exposure to stepwise increasing concentrations of camptothecin up to 10 ng ml$^{-1}$ ($C6_{CPT10}$), 50 ng ml$^{-1}$ ($C6_{CPT50}$) and 100 ng ml$^{-1}$ ($C6_{CPT100}$), these concentrations being then maintained in the cell culture medium. Table 1 presents the $IC_{50}$ of several anticancer drugs in these lines. The $C6_{CPT10}$ line is characterised by a 15-fold resistance to camptothecin and a collateral sensitivity to etoposide (3.6-fold) without any change in doxorubicin cytotoxicity and a slight resistance to vincristine. The $C6_{CPT50}$ and $C6_{CPT100}$ cell lines are characterised by a 30-fold resistance to camptothecin and a slight cross resistance to both top 2 inhibitors and to vincristine.

Top 1, top 2α and P-glycoprotein were quantified by immunoblotting of cellular extracts (Figure 1). There was a significant 50% decrease in top 1 amount in $C6_{CPT10}$ and a 70% decrease in $C6_{CPT50}$ and $C6_{CPT100}$ cells. In contrast, there was a 2-fold increase in top 2α level in $C6_{CPT50}$ and $C6_{CPT100}$ cells and no significant change in its level in $C6_{CPT10}$ cells.

Catalytic activities of top 1 and top 2 were studied in C6, $C6_{CPT10}$ and $C6_{CPT50}$ cells (Table 2). There was a decrease in top 1 relaxation activity in both camptothecin-resistant cell lines as compared to the C6 cell line, and a significant increase in top 2 decatenation activity occurring only in the $C6_{CPT50}$ line.

Camptothecin accumulation was evaluated in C6, $C6_{CPT10}$ and $C6_{CPT50}$ cells by HPLC using fluorescence detection. After 4-hour incubations at the concentration of 50 µM, no significant difference in drug accumulation was observed between the 3 cell lines (0.242 ± 0.20 nmoles per $10^6$ cells in wild-type cells versus 0.222 ± 0.22 in $C6_{CPT10}$ cells and 0.206 ± 0.20 in $C6_{CPT50}$ cells).

### Cytotoxicity of combinations of camptothecin and top 2 inhibitors

The simultaneous use of a top 1 and a top 2 inhibitor at a fixed molar ratio over a large range of concentrations allowed the evaluation of their possible synergistic or antagonistic effects on cell survival of the various C6 cell lines. Plotting log (1–SF/SF) as a function of log (dose) gave in all cases a linear regression coefficient

**Table 1** Cytotoxicity of various anticancer drugs towards the C6 cell variants

| | $IC_{50}$ of the anticancer drugs tested (nM) | | | |
|---|---|---|---|---|
| | Camptothecin | Doxorubicin | Etoposide | Vincristine |
| C6 | 61.1 ± 11.2 | 21.2 ± 2.1 | 207 ± 21 | 3.7 ± 0.7 |
| $C6_{CPT10}$ | 890 ± 31 | 20.9 ± 7.4 | 56.9 ± 3.4 | 10.6 ± 1.8 |
| $C6_{CPT50}$ | 1596 ± 116 | 94.5 ± 5.7 | 366 ± 26 | 31.4 ± 2.4 |
| $C6_{CPT100}$ | 1866 ± 72 | 88.4 ± 16 | 334 ± 13 | 25.5 ± 1.4 |

*© 2001 Cancer Research Campaign* 

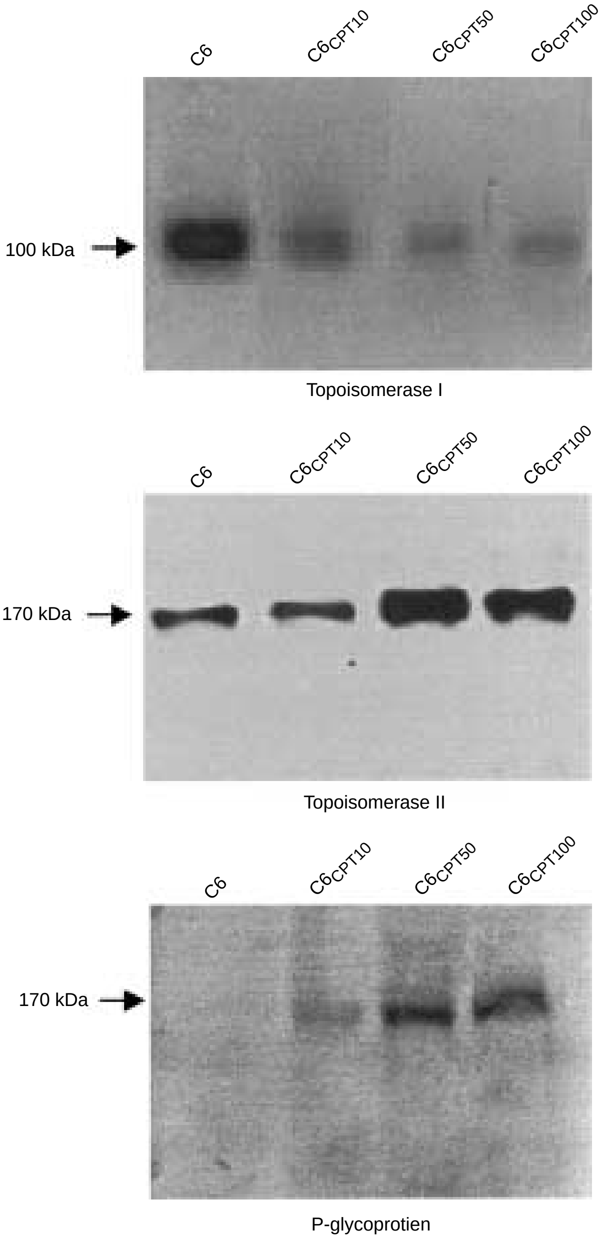

**Figure 1** Western blots of the nuclear extracts (for top 1 and top 2α) and of total cell lysates (for P-glycoprotein) of C6 cells and camptothecin-resistant variants. Extracts were submitted to electrophoresis and blotting as described in Material and Methods. Revelation was obtained after incubation with specific primary antibodies followed by antibody detection

**Table 2** Catalytic activities of topoisomerases I and II in C6 cells and camptothecin-resistant variants

| | Catalytic activity | |
|---|---|---|
| | **Topoisomerase I** | **Topoisomerase II** |
| C6 | 7.03 ± 2.38 | 65.1 ± 2.3 |
| $C6_{CPT10}$ | 2.58 ± 1.02* | 70.5 ± 2.3* |
| $C6_{CPT50}$ | 1.03 ± 0.24** | 93.4 ± 1.8*** |

Results are expressed as ng DNA relaxed per ng protein (top 1) or as ng DNA decatenated per μg protein (top 2). Significance as follows: *:$P < 0.05$; **:$P < 0.01$; ***:$P < 0.001$.

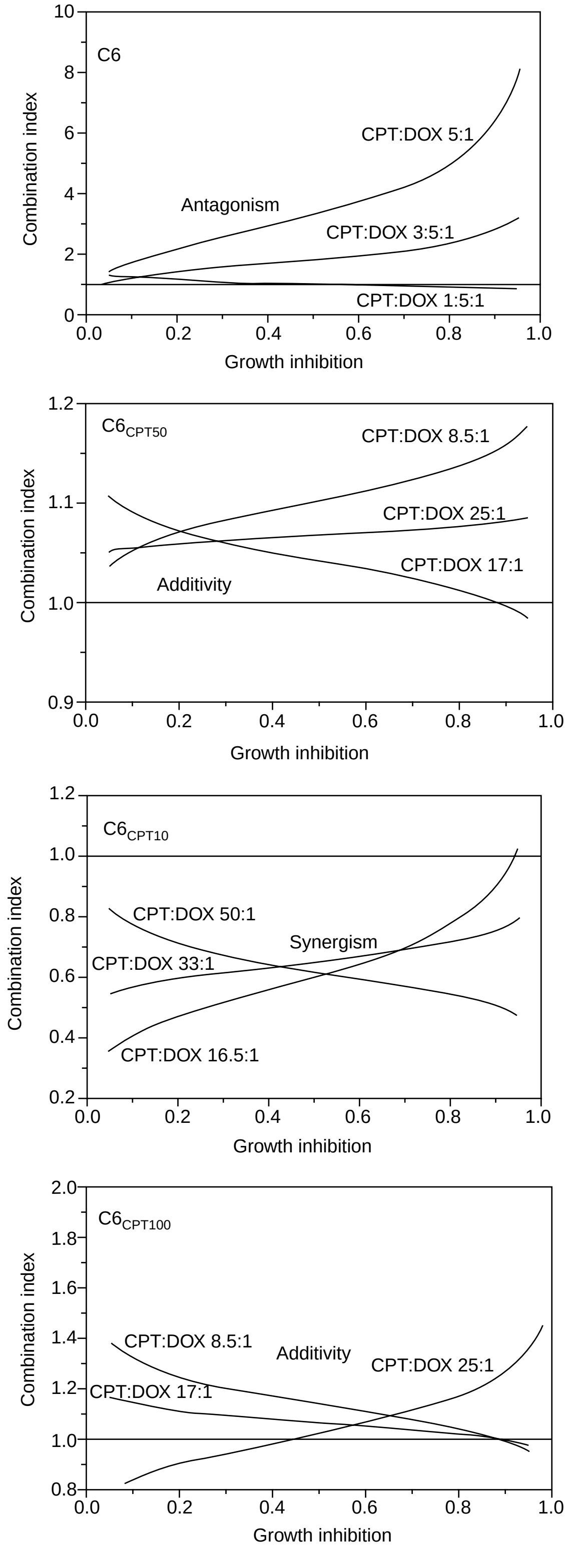

**Figure 2** Analysis of combined effects of camptothecin and doxorubicin administered simultaneously as 24-h exposures to C6 cells and camptothecin-resistant variants. The dose ratios used for cytotoxicity experiments are indicated on the graphs. Using the Chou and Talalay (1984) median-effect principle revealed, at the $IC_{50}$s of the combinations, synergism (combination index < 1), additivity (combination index not significantly different from 1) or antagonism (combination index > 1) as indicated. Note that the ordinate scales are different for each graph

 *© 2001 Cancer Research Campaign*

≥ 0.95, which justifies the use of the median-effect principle for the evaluation of synergistic or antagonistic interactions.

Simultaneous exposures to camptothecin and doxorubicin appeared additive or antagonistic in the C6 line (Figure 2), with the antagonistic effect increasing as a function of the increase in the proportion of camptothecin in the mixture. This association was clearly synergistic in the $C6_{CPT10}$ line but was never more than additive in the $C6_{CPT50}$ and the $C6_{CPT100}$ cell lines.

Simultaneous exposures to camptothecin and etoposide were strictly additive in the C6 cell line (Figure 3). In the $C6_{CPT10}$ cell line, the combination was slightly but consistently synergistic but additivity only was obtained in the $C6_{CPT50}$ and $C6_{CPT100}$ cell lines. In all cell lines, sequential exposures to camptothecin and doxorubicin appeared additive when camptothecin was given first and antagonistic when doxorubicin was given first (Figure 4).

### DNA damage induced by camptothecin and etoposide in the C6 cell line

The DNA damage induced by etoposide, alone and in combination with camptothecin, was evaluated in the C6 cell line as a complement, at the molecular level, of the cytotoxicity experiments. With etoposide at 1 and 2 μM, there was a concentration-dependent increase in DNA damage, from 0.94 ± 0.17 to 1.62 ± 0.15 Gy equivalents. The combination of etoposide and camptothecin, both used at 1 μM concentration, induced DNA damage equivalent to that obtained with etoposide at 2 μM (1.53 ± 0.06 Gy equivalents), corresponding well with the additive cytotoxicity observed in this cell line at equimolar concentrations.

### DISCUSSION

The cell lines we selected for resistance to camptothecin display in fact several alterations contributing to a complex phenotype of cross-resistance. All of them display a reduction in top 1 content and catalytic activity, a feature relatively frequent in cell lines selected for resistance to top 1 inhibitors (Andoh and Okada, 1994). These cell lines present in addition 2 alterations which appear related to top 2 – interfering drugs: an increase in the level of top 2α and increased catalytic activity of top 2, generally considered as leading to an increased sensitivity to drugs such as etoposide and doxorubicin (Robert and Larsen, 1998), and an overexpression of P-glycoprotein, which in contrast contributes to resistance to the same drugs (Endicott and Ling, 1989).

The development of these 2 opposite mechanisms probably involves 2 independent phenomena. On the one hand, P-glycoprotein has been consistently found to be overexpressed in response to various kinds of stress, such as irradiation (Hill et al, 1990) or cytotoxic compounds not even expelled by this pump (Chaudhary and Roninson, 1993). In our camptothecin-resistant cell lines, there is a slight increase in P-glycoprotein expression at the first level of selection (probably responsible for the 3-fold resistance of this cell line to vincristine) and an important increase in this expression in the most resistant lines, $C6_{CPT50}$ and $C6_{CPT100}$. Despite this overexpression, the $C6_{CPT50}$ line presents no reduced accumulation of camptothecin after 4 hours of incubation with this drug, confirming that camptothecin is not a substrate for P-glycoprotein (Chen et al, 1991).

On the other hand, the increase in top 2α level observed in the most resistant cell lines, as well as the increase in catalytic activity also detected in all resistant lines, is a feature that has been already

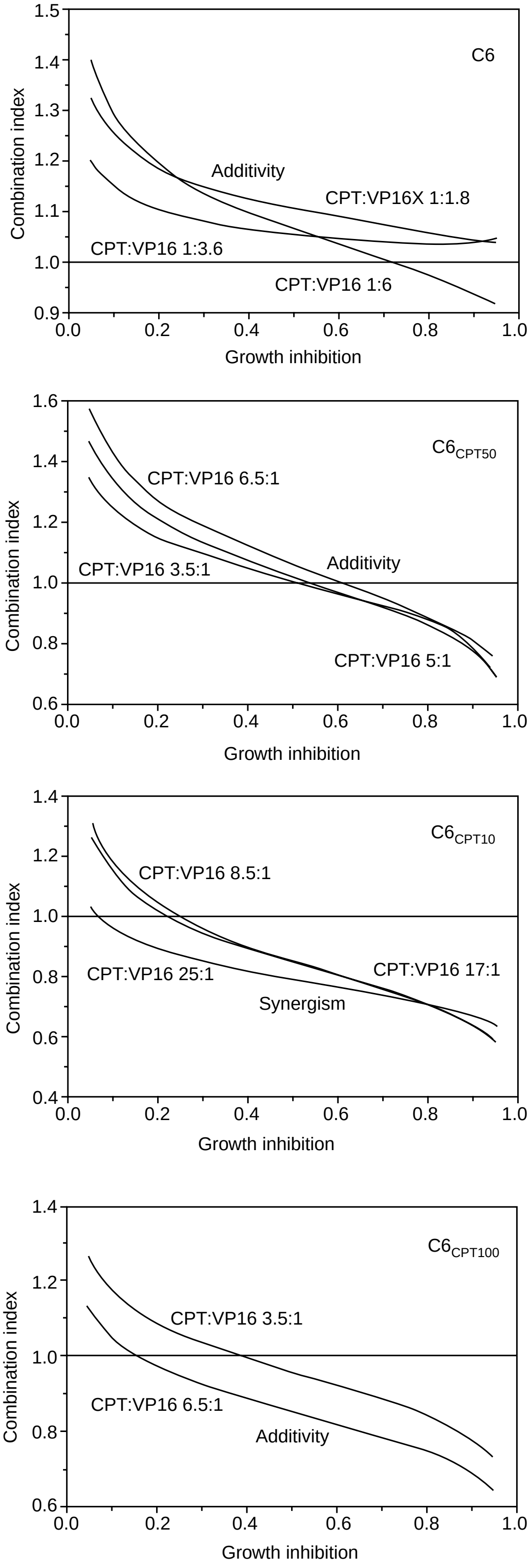

**Figure 3** Analysis of combined effects of camptothecin and etoposide administered simultaneously to C6 cells and camptothecin-resistant variants. Same legend as Figure 2

*© 2001 Cancer Research Campaign*

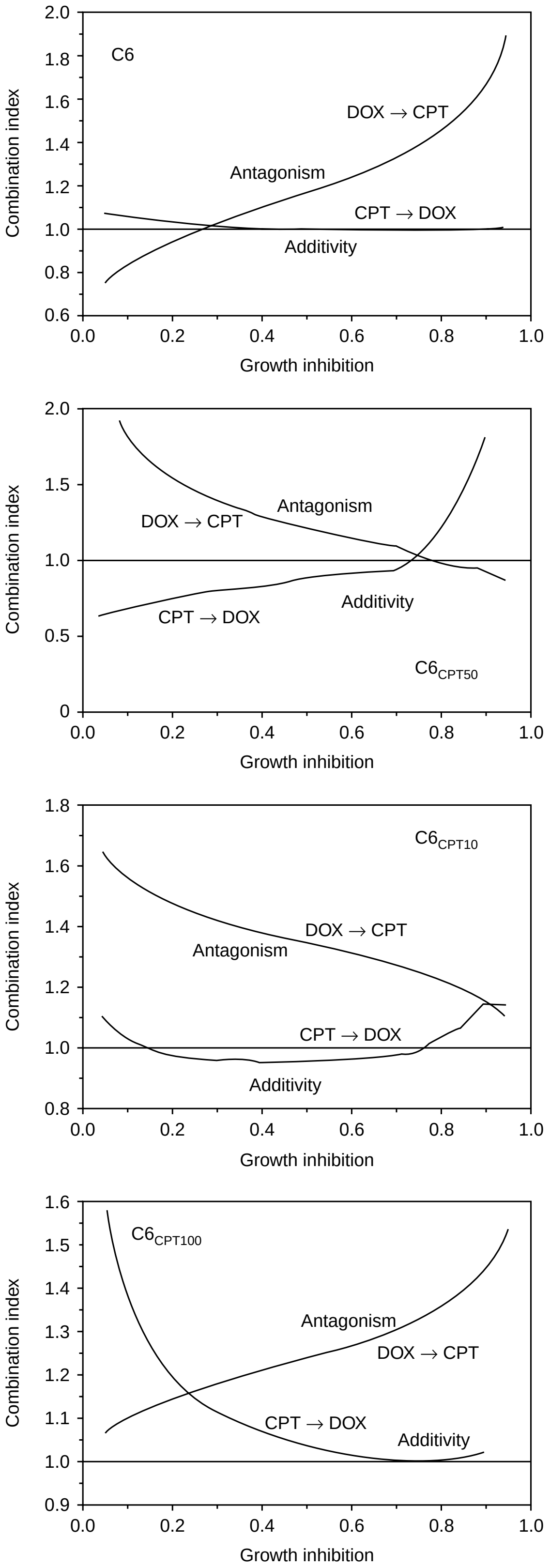

**Figure 4** Analysis of combined effects of camptothecin and doxorubicin administered sequentially to C6 cells and camptothecin-resistant variants. The sequence of the 24-h exposures to each drug is indicated on the graphs. Same legend as Figure 2

observed in cell lines selected with top 1 inhibitors (Sugimoto et al, 1990). It has been interpreted as a regulatory mechanism for compensating the top 1 down-regulation in such cell lines. This slight, but significant increase in top 2α activity is likely to be responsible for the significant hypersensitivity of the $C6_{CPT10}$ cell line to etoposide and the unchanged sensitivity of this line to doxorubicin. In this line, the level of expression of P-glycoprotein would be too low to exert an important effect on the efflux of these drugs, in addition to the fact that etoposide is a relatively poor substrate for P-glycoprotein. In the $C6_{CPT50}$ and $C6_{CPT100}$, the P-glycoprotein-mediated drug efflux would be enough to counteract the collateral intrinsic sensitivity to etoposide and doxorubicin due to top 2α overexpression.

Such complex drug-resistant phenotypes, implying concomitant alterations of several determinants of drug activity, is frequently observed during the selection process of resistant cells in vitro (‘multifactorial’ resistance, Rabier et al (1991) and renders relatively complex the interpretation of cytotoxicity data obtained during drug combinations. In our cell models, the combination of a top 1 inhibitor (camptothecin) and a top 2 inhibitor (etoposide or doxorubicin) is not more than additive in wild-type cells. A slight, but significant synergistic effect was observed in the $C6_{CPT10}$ cell line when doxorubicin or etoposide were combined with camptothecin, using several drug ratios corresponding to the ratio of the respective $IC_{50}$s of the drugs in the different cell lines. This synergistic effect can be interpreted as a consequence of the increased top 2 activity in this cell line. In contrast, in the $C6_{CPT50}$ and the $C6_{CPT100}$ lines, this synergy was lost, as P-glycoprotein prevents the top 2 increase to sensitise the cells to doxorubicin or etoposide.

Several studies have compared the effect of combinations of top 1 and top 2 inhibitors in different types of cell lines. Kaufmann (1991) observed an important antagonism exerted by camptothecin on the cytotoxicity of etoposide in HL 60 cells. Most of the subsequent studies found at best an additive effect between camptothecin (or a camptothecin analogue such as SN-38 or topotecan) and etoposide or doxorubicin (Bertrand et al, 1992; Stahl et al, 1997), but a schedule-dependency was sometimes observed: when the top 1 inhibitor was administered before the top 2 inhibitor, a synergy could be observed (Masumoto et al, 1995; Bonner and Kozelsky, 1996). In the present study, such combinations appeared synergistic only in the $C6_{CPT10}$ cell line, the only one presenting simultaneously no major change in top 2 content and activity, and a quite moderate P-glycoprotein expression. In the other cell lines, combinations of camptothecin and doxorubicin or etoposide appeared generally additive. In the C6 cell line, combinations of camptothecin and doxorubicin were even antagonistic whereas combinations of camptothecin and etoposide were additive. It must be kept in mind that doxorubicin is an intercalator acting at the level of the cleavable complex, whereas etoposide does not directly interfere with DNA. The steric hindrance resulting from the concomitant presence of doxorubicin and camptothecin on the same DNA sites might explain why their combinations are antagonistic.

This in vitro evaluation of combinations of a top 1 and a top 2 inhibitor does not allow to predict what would be the results of such combinations in vivo or in the clinical setting. However, it suggests that combining top 1 and top 2 inhibitors might be more interesting in documented situations of resistance to top 1 inhibitors *not* accompanied by P-glycoprotein overexpression. The narrow therapeutic window offered for such combinations may,

 *© 2001 Cancer Research Campaign*

therefore, not be exploitable in the clinical setting, for which synergistic combinations are desired, and even expected, when combining inhibitors of the 2 types of topoisomerase. Up to now, clinical combinations of top 1 and top 2 inhibitors have not been studied in detail, since only phase I reports have been published to date, not allowing the evaluation of these combinations (Ando et al, 1997; Herben et al, 1997).

### ACKNOWLEDGEMENTS

This work was supported by grants from the Ligue Nationale contre le Cancer, Comités de la Charente et de la Charente-Maritime.

### REFERENCES

Andersen AH, Svejstrup JQ and Westergaard O (1994) The DNA binding, cleavage, and religation reactions of eukaryotic topoisomerases I and II. *Adv Pharmacol* **29A**: 83–101

Ando M, Eguchi K, Shinkai T, Tamura T, Ohe Y, Kurata T, Ohmatsu H, Kubota K, Sekine I, Hojo N, Matsumoto T, Kodama T, Klakinuma R, Nishiwaki Y and Saijo N (1997) Phase I study of sequentially administered topoisomerase I inhibitor (irinotecan) and topoisomerase II inhibitor (etoposide) for metastatic non-small cell lung cancer. *Br J Cancer* **76**: 1494–1499

Andoh T and Okada K (1994) Drug resistance mechanisms of topoisomerase I drugs. *Adv Pharmacol* **29B**: 93–103

Benda P, Lightbody J, Sato G, Levine L and Sweet W (1968) Differentiated rat glial cell strain in tissue culture. *Science* **161**: 370–371

Bertrand R, O'Connor PM, Kerrigan D and Pommier Y (1992) Sequential administration of camptothecin and etoposide circumvents the antagonistic cytotoxicity of simultaneous drug administration in slowly growing human colon carcinoma HT-29 cell. *Eur J Cancer* **28**: 743–748

Bonner JA and Kozelsky TF (1996) The significance of the sequence of administration of topotecan and etoposide. *Cancer Chemother Pharmacol* **39**: 109–112

Chaudhary PM and Roninson IB (1993) Induction of multidrug resistance in human cells by transient exposure to different chemotherapeutic drugs. *J Nat Cancer Inst* **85**: 632–639

Chen AY, Yu C, Potmesil M, Wall ME, Wani MC and Liu LF (1991) Camptothecin overcomes MDR1-mediated resistance in human KB carcinoma cells. *Cancer Res* **51**: 6039–6044

Chou J and Chou TC (1987) Dose-effect analysis with microcomputers. Biosoft: Cambridge

Chou TC and Talalay P (1984) Quantitative analysis of dose-effect relationships: the combined effect of multiple drugs or enzyme inhibitors. *Adv Enzyme Regul* **22**: 27–55

Endicott JA and Ling V (1989) The biochemistry of P-glycoprotein-mediated multidrug resistance. *Annu Rev Biochem* **58**: 137–171

Herben VMM, ten Bokkel Huinink WW, Dubbelman AC, Mandjes IAM, Groot Y, van Gortel-van Zomeren DM and Beijnen JH (1997) Phase I and pharmacological study of sequential intravenous topotecan and oral etoposide. *Br J Cancer* **76**: 1500–1508

Hill BT, Deuchars K, Hosking LK, Ling V and Whelan RDH (1990) Overexpression of P-glycoprotein in mammalian tumor cell lines after fractionated X irradiation in vitro. *J Nat Cancer Inst* **82**: 607–612

Huet S, Schott B and Robert J (1992) P-glycoprotein overexpression cannot explain the complete doxorubicin resistance phenotype in rat glioblastoma cell lines. *Br J Cancer* **65**: 538–544

Kaufmann SH (1991) Antagonism between camptothecin and topoisomerase II-directed chemotherapeutic agents in a human leukemia cell line. *Cancer Res* **51**: 1129–1136

Kohn KW (1991) Principles and practice of DNA filter elution. *Pharmacol Ther* **49**: 55–77

Laemmli UK (1970) Cleavage of structural proteins during the assembly of the head of bacteriophage T4. *Nature* **227**: 680–685

Liu LF (1989) DNA topoisomerase poisons as antitumor drugs. *Annu Rev Biochem* **58**: 351–375

Liu LF and Miller KG (1981) Eukaryotic DNA topoisomerases: two forms of type I DNA topoisomerases from HeLa cell nuclei. *Proc Natl Acad Sci USA*: 3487–3491

Masumoto N, Nakano S, Esaki T, Tatsumoto T, Fujishima H, Baba E, Nakamura M and Niho Y (1995) Sequence-dependent modulation of anticancer drug activities by 7-ethyl-hydroxy-camptothecin in an HST-1 human squamous carcinoma cell line. *Anticancer Res* **15**: 405–410

Montaudon D, Pourquier P, Denois F, de Tinguy-Moreaud E, Lagarde P and Robert J (1997) Differential stabilization of topoisomerase II – DNA cleavable complexes by doxorubicin and etoposide in doxorubicin-resistant rat glioblastoma cell. *Eur J Biochem* **245**: 307–315

Nitiss JL (1994) Roles of DNA topoisomerases in chromosomal replication and segregation. *Adv Pharmacol* **29A**: 103–134

Oguro M, Seki Y, Okada K and Andoh T (1990) Collateral drug sensitivity induced in CPT-11 (a novel derivative of camptothecin)-resistant cell lines. *Biomed Pharmacother* **44**: 209–216

Osheroff N, Corbett AH and Robinson MJ (1994) Mechanism of action of topoisomerase II – targeted antineoplastic drugs. *Adv Pharmacol* **29B**: 105–126

Pommier Y, Tanizawa A and Kohn KW (1994) Mechanisms of topoisomerase I inhibition by anticancer drugs. *Adv Pharmacol* **29B**: 73–92

Rabier MJ, Bruno NA and Slate DL (1991) Multifactorial resistance in LS174T human colon carcinoma cells selected with doxorubicin. *Int J Cancer* **49**: 601–607

Robert J and Larsen AK (1998) Drug resistance to topoisomerase II inhibitors. *Biochimie* **80**: 247–254

Stahl M, Kasimir-Bauer S and Harstrick (1997) Down-regulation of topoisomerase II by camptothecin does not prevent additive activity of the topoisomerase II inhibitor etoposide in vitro. *Anticancer Drug* **8**: 671–676

Sugimoto Y, Tsukahara S, Oh-hara T, Liu LF and Tsuruo T (1990) Elevated expression of DNA topoisomerase II in camptothecin-resistant human tumor cell lines. *Cancer Res* **50**: 7962–7965

Tan KB, Mattern MR, Eng WK, McCabe FL and Johnson RK (1989) Nonproductive rearrangement of DNA topoisomerase I and II genes: correlation with resistance to topoisomerase inhibitors. *J Nat Cancer Inst* **81**: 1732–1735

Wang JC (1994) DNA topoisomerases as targets of therapeutics. An overview. *Adv Pharmacol* **29A**: 1–19

*© 2001 Cancer Research Campaign*
